# Activity Dependent Modulation of Granule Cell Survival in the Accessory Olfactory Bulb at Puberty

**DOI:** 10.3389/fnana.2017.00044

**Published:** 2017-05-23

**Authors:** Livio Oboti, Sara Trova, Roberta Schellino, Marilena Marraudino, Natalie R. Harris, Olubukola M. Abiona, Mojca Stampar, Weihong Lin, Paolo Peretto

**Affiliations:** ^1^Center for Neuroscience Research, Children’s National Health System, WashingtonDC, United States; ^2^Department of Life Sciences and Systems Biology, Neuroscience Institute Cavalieri Ottolenghi, University of TorinoOrbassano, Italy; ^3^Department of Neurosciences “Rita Levi Montalcini”, University of TurinTurin, Italy; ^4^Department of Biological Sciences, University of Maryland, Baltimore County, BaltimoreMD, United States; ^5^Research Center for Genetic Medicine, Children’s National Health System, WashingtonDC, United States

**Keywords:** puberty, vomeronasal system, neurogenesis, sensory adaptation, sexual signals

## Abstract

The vomeronasal system (VNS) is specialized in the detection of salient chemical cues triggering social and neuroendocrine responses. Such responses are not always stereotyped, instead, they vary depending on age, sex, and reproductive state, yet the mechanisms underlying this variability are unclear. Here, by analyzing neuronal survival in the first processing nucleus of the VNS, namely the accessory olfactory bulb (AOB), through multiple bromodeoxyuridine birthdating protocols, we show that exposure of female mice to male soiled bedding material affects the integration of newborn granule interneurons mainly after puberty. This effect is induced by urine compounds produced by mature males, as bedding soiled by younger males was ineffective. The granule cell increase induced by mature male odor exposure is not prevented by pre-pubertal ovariectomy, indicating a lesser role of circulating estrogens in this plasticity. Interestingly, the intake of adult male urine-derived cues by the female vomeronasal organ increases during puberty, suggesting a direct correlation between sensory activity and AOB neuronal plasticity. Thus, as odor exposure increases the responses of newly born cells to the experienced stimuli, the addition of new GABAergic inhibitory cells to the AOB might contribute to the shaping of vomeronasal processing of male cues after puberty. Consistently, only after puberty, female mice are capable to discriminate individual male odors through the VNS.

## Introduction

Postnatal development is a critical period for the maturation of brain circuits and implies extensive interactions between external and internal factors affecting both their wiring and tuning. This is particularly important for the brain systems implied in goal oriented behaviors (such as mating or foraging) as they need to adaptively match external stimuli to internal states and drives, in order to limit non-adaptive decisions that could be detrimental for survival (Nowicki et al., 2002; Ramm et al., 2008; Bouret, 2012; Saleem et al., 2014). Because mating and feeding circuits have been mainly considered hard-wired or innate (Anderson, 2016; Kohl et al., 2017), the neural mechanisms involved in this tuning remain poorly investigated. Here, we have analyzed the role of sensory activity in the peripubertal activity-dependent maturation of inhibitory interneurons in the accessory olfactory bulb (AOB), a bulbar circuit specialized in the elaboration of salient signals for reproductive and mating behaviors (Mucignat-Caretta et al., 1995; Keller et al., 2006; Hendrickson et al., 2008; Brennan, 2009; Haga et al., 2010; Flanagan et al., 2011; Ferrero et al., 2013). The onset of female puberty represents a critical period for the development of both reproductive organs and brain circuits regulating the neuroendocrine aspects of sexual behavior (Romeo, 2003). Also, puberty represents a stage for important behavioral changes (Sisk and Zehr, 2005; Blakemore et al., 2010) and a critical period for the activity dependent remodeling of cortical circuits (Piekarski et al., 2017). In mice, at puberty chemical signaling begins to be used to advertise sexual receptivity and to assess both the quality and readiness of mating partners (Moncho-Bogani et al., 2002; Ramm et al., 2008). These two aspects of sexual maturation – the internal physiology and the sensitivity to environmental cues – obviously interact, and, through the vomeronasal system (VNS), chemical stimuli can affect both the development (Vandenbergh, 1967, 1974; Colby and Vandenbergh, 1974; Flanagan et al., 2011; Oboti et al., 2014) and the adult display (Haga et al., 2010; Roberts et al., 2010; Ferrero et al., 2013; Oboti et al., 2014) of female reproductive behaviors.

A tight interaction between neuroendocrine responses and the molecular nature of specific triggering signals has long been considered the main mechanism by which both aspects of sexual maturation are matched. However, chemical signaling is flexible and subjected to changes depending on both external and internal factors (Drickamer, 1992; Lemmen and Evenden, 2009; Garratt et al., 2011; Ferrero et al., 2013; Villar et al., 2015). In fact, the same compounds might acquire different signaling values depending on behavioral and physiological needs (Mucignat-Caretta et al., 1998; Brennan, 2009; Martín-Sánchez et al., 2015; Stowers and Liberles, 2016). Therefore, the question of how the VNS determines the onset of social behavioral responses by detecting such variable signals is unclear. Clarifying this issue is further complicated by the presence of ongoing neurogenesis in both the vomeronasal epithelia (Weiler, 2005) and the AOB (Oboti et al., 2009; see for a review Oboti and Peretto, 2014). Specifically, how this plasticity relates to the onset of puberty and to more mature behavioral displays is still unclear.

In our previous works we have extensively characterized the process of adult neurogenesis in the AOB, showing that, as in the main olfactory bulb (MOB), adult neurogenesis in this region involves a process of continuous addition of GABAergic interneurons originating in the subventricular zone (SVZ; Bonfanti et al., 1997; Peretto et al., 2001; Oboti et al., 2009, 2011). In addition, we have also shown that the integration and survival of AOB immature neurons is modulated by sensory activity (Oboti et al., 2009, 2011). In particular, exposure to vomeronasal organ (VNO)-detected stimuli contained in male urine, during a critical stage of newborn cell maturation, increases neuronal survival in the AOB of female mice (Oboti et al., 2011).

Here, by incorporating bromodeoxyuridine (BrdU) in immature SVZ-derived neurons reaching the AOB, we show that granule cell (GC) survival in the female AOB begins to be particularly sensitive to opposite-sex olfactory stimuli at the onset of puberty. We further show that the number of GCs integrating in the AOB is modulated by olfactory stimuli derived from adult but not juvenile male urine. Moreover, short term post-pubertal exposure of females to adult male derived cues promotes the integration of immature GCs in the bulbar circuits implied in male odor processing. This further correlates with the onset of individual specific responses to male odors detected through the VNS, normally displayed by mature females (Moncho-Bogani et al., 2002; Ramm et al., 2008). By combining ovariectomy with male odor exposure in BrdU-treated animals, we next provide evidence supporting that the effect of male derived cues on AOB GCs does not depend on ovarian hormones. Rather, increased VNO activity levels shown by post-pubertal females in response to these stimulations suggest a major role of sensory activity in this process. Overall, our results indicate that through activity dependent regulation of AOB GC development, VNO- detected stimuli rearrange the AOB inhibitory network involved in their processing, when these cues acquire the meaning of mating signals after puberty.

## Materials and Methods

### Animals

All experiments were done using CD-1 mice (Charles River, Italy): females and males, pre-pubertal (postnatal day P21–P28), peri-pubertal (P35–P42), and post-pubertal (P52–P90). Animals were housed under a 12-h light-dark cycle in an environmentally controlled room. Female subjects of the same group were maintained 4–6 per cage, whereas male subjects were kept or in isolation for the duration of all experiments or in cages with other male subjects of the same group. Experimental procedures were in accordance with the European Communities Council Directive of 24 November 1986 (86/609/EEC), Recommendation 18/06/2007, Dir. 2010/63/UE, and the Italian law for care and use of experimental animals (DL116/92) and were approved by the Italian Ministry of Health and the Bioethical Committee of the University of Turin (Protocol Number DGSAF0007085-A05/04/2013). All animal care and procedures conducted at the University of Maryland, Baltimore County were in accordance with the National Institutes of Health guide for the Care and Use of Laboratory Animals (2006) and approved by the institutional Animal Care and Use Committee. All experiments were designed to minimize the number of animals used.

### Assessment of Puberty Onset

CD1 female mice were weaned at P21 and checked daily for vaginal opening. After vaginal opening, vaginal smears were performed daily and analyzed under an inverted microscope to identify the specific day of first estrus.

### BrdU Treatment and Bedding Exposure

To identify newly generated cells in the AOB, female mice were intraperitoneally injected with BrdU in 0.1 M Tris (pH 7.4) twice in 1 day (delay = 8 h, 100 mg/kg body weight), and sacrificed 28 days later for the evaluation of neuronal survival. For all experimental groups, bedding stimulations (consisting of daily renewed male bedding material added with male urine) were timed during the 2nd week after BrdU injection. Males were caged individually or in groups of 3–4 animals and the bedding was left untouched for at least 1 week before being transferred to female cages. Exposures were performed at different stages of peripubertal maturation: from P28 to P35 (pubertal); from P35 to P42 (post-pubertal) and from P52 to P59 (mature). Different sources of male soiled-bedding material were used for each experimental group. Females exposed to sexually mature males, were given a mixture of bedding from three different adult reproductive males not kin-related with females (MB); females exposed to coetaneous males bedding, were given a mixture of bedding from 3/4 kin-unrelated males of the same age of female subjects (cMB; P28–P35 and P35–P42 for the peripubertal exposure; P52–P59 for the adult exposure); females exposed to kin or foster males (of the same age as females) bedding, were given a mixture from 3/4 kin (kMB) or foster (foMB) (cross-foster with females at the age of P2 until P21) males, respectively. The “control” groups were treated similarly but with clean bedding.

### Ovariectomy

Juvenile (P21, *n* = 7) CD1 female mice were deeply anesthetized with a 3:1 solution of ketamine (Ketavet; Gellini, Italy) and xylazine (Rompun; Bayer, Germany) and using aseptic procedures both ovaries were removed by two small incisions on each side in the abdominal area, one through the skin and then another through the muscle wall. Each ovary was tied off with absorbable surgical thread and removed; after that the muscle incision and skin incision were closed using sutures. Sham-operated juvenile (P21, *n* = 6) CD1 female mice have been subjected to same surgical manipulations without removal of the ovaries. Mice were then allowed to recover for 1 week before further treatments.

### VNO Dye-Access Assay

Juvenile (3 weeks old) sexually naïve CD1 female mice were purchased from the Jackson Laboratory and grown to designed ages of either 28, 35, or 52 days old, respectively (*n* = 5–7 per group).

#### Urine Collection and Urine-Dye Mixture

Male urine was freshly collected on the same day of the experiment from the same males that were sexually experienced. To induce urination from the males, we placed two female CD1 mice into two clean separate cages without bedding for 2 min. We then removed the female mice and transferred the male mice into the scented cages. Care was taken to remove female urine or feces, if any, before introducing the males into the cages. If no urine appeared after 2 min, the procedure was repeated or the animal was held by hand and the bladder region gently pressed to induce urination. Male urine was immediately collected after urination using a pipette. Urine was pooled into an Eppendorf tube, and placed on ice before use. For the stimulus-dye VNO access assay, a 100 μl of urine was mixed with Rhodamine 6G fluorescent dye (Sigma) to yield a final concentration of urine in 1:10 dilution and 0.002% dye.

#### Delivery of Urine-Dye Mixture

The VNO dye access assay was performed following a previously developed method from Ogura et al. (2010). Briefly, an individual mouse was transferred and acclimated in a chamber made from clean empty, plastic, pipette tip boxes with clear lids 12.7 cm × 7.6 cm × 5.1 cm (width × depth × height) with a 1.27 cm square hole on a side wall for 10 min. Upon acclimation, a pipette tip containing 5 μl of urine-dye solution was presented to the nostrils in small drops, when the mouse protruded its nose from the small hole. The process was repeated until all 5 μl was delivered. The process usually lasted 3–10 min. For each post-developmental stage, five to seven mice were examined.

#### VNO Dissection and Fluorescence Image Acquisition

Immediately after the assay, mice were euthanized by CO_2_ inhalation followed by cervical dislocation. The head was then removed, split roughly along the midline to expose the VNO. The half head containing the VNO was placed on a premade mold and epi-fluorescence images were taken for both lateral and ventral VNO using a 4× lens and an Olympus BX 41 epi-fluorescence compound microscope equipped with a Retiga 4000R camera and Image-Qcapture Pro 7 (QImaging, British Columbia, Canada). Fluorescence dye intensity of the VNO proper and entrance duct, which is narrow, approximately 0.4 mm in length, tubular structure anterior to the VNO proper (Ogura et al., 2010), were measured using NIH ImageJ. Background intensity was measured from the septal respiratory epithelium dorsal to the VNO proper.

### Tissue Preparation for Immunohistochemistry

Mice were deeply anesthetized with an intraperitoneal injection of ketamine (Ketavet; Gellini, Italy) and xylazine (Rompun; Bayer, Germany) 3:1 solution. All the animals were transcardially perfused with 0.9% saline solution followed by cold 4% paraformaldehyde (PFA) in 0.1 M phosphate buffer (PB), pH7.4. Brains were removed from skull and post-fixed for 4 h in 4% PFA at 4°C, followed by a cryopreservation step using a 30% sucrose solution in 0.1 M PB pH 7.4 at 4°C. The two hemispheres were separated and embedded in OCT (Bio-Optica), and then frozen and cryostat-sectioned. Free floating parasagittal sections (25 μm) were collected in multi-well dishes in representative series of the AOB. Sections were stored at -20°C in an antifreeze solution (30% ethylene glycol, 30% glycerol, 10% phosphate buffer: 189 mM NaH_2_PO_4_, 192.5 mM NaOH; pH 7.4) until use.

### Immunohistochemistry

After rinsing in phosphate-buffered saline (PBS) to remove the antifreeze solution, sections were incubated for 24 h at 4°C in primary antibodies diluted in 0.01 M PBS, pH 7.4, 0.5% TritonX-100, and 1% normal sera, made in the same host species of the secondary antibodies. As primary antibodies we used: anti-BrdU, rat IgG monoclonal, dilution 1:5000 (ABC), AbD serotec, Bio-Rad Laboratories, code number OBT0030CX (Liu et al., 2009); anti-c-Fos, rabbit IgG polyclonal, dilution 1:10000 (IFL), Santa Cruz Biotechnologies, Santa Cruz, CA, United States, code number sc52 (Oboti et al., 2011); anti-NeuN, mouse IgG monoclonal, dilution 1:1000 (IFL), Chemicon International, Temecula, CA, United States, code number MAB377 (Oboti et al., 2009); anti-GAD67, dilution 1:1000 (IFL), mouse IgG monoclonal, Chemicon International, Temecula, CA, United States, code number MAB5406 (Oboti et al., 2009). For BrdU immunostaining, sections were pre-treated with 2 N HCl for 30 min at 37°C, for antigene retrieval, and neutralized with borate buffer, pH 8.5, for 10 min. For the avidin-biotin-peroxidase method, sections were incubated for 1 h at room temperature in biotinylated secondary antibody (anti-rat IgG; Vector Laboratories, Burlingame, CA, United States) diluted 1:250 in 0.01 M PBS, pH 7.4 following by avidin-biotin-peroxidase complex (Vector Laboratories). To reveal immunoreactivity, we used 0.015% 3,3′-diaminobenzidine and 0.0024% H_2_O_2_ in 0.05 M Tris-HCl, pH 7.6. After adhesion on gelatin-coated glass slides, sections were mounted in DPX (Merck-Millipore, VWR International PBI, Milan, Italy). For immunofluorescence double-staining, the sections were incubated in a mixture of primary antibodies and appropriate blocking sera for 24 h at 4°C, then incubated with appropriate fluorochrome-conjugated secondary antibodies (Cy3-conjugated secondary Ab, 1:800; 488-conjugated secondary Ab, 1:400; Jackson ImmunoResearch Laboratories, United States) and/or biotinylated secondary antibodies (1:250; Vector Laboratories, Burlingame, CA, United States), and finally incubated with avidin-FITC (1:400, Vector Laboratories, Burlingame, CA, United States). The sections were then coverslipped with the anti-fade mounting medium Dabco (Sigma) and analyzed with a laser scanning LAS AF Lite confocal system (Leica Microsystems).

### Cell Counting and Statistical Analysis

The number of BrdU-positive nuclei in the AOB GC layer was established by counting peroxidase/DAB-stained 25 μm thick parasagittal sections in all experimental groups. Three series (*n* = 7 sections/animal) representing the whole AOB were used for each animal. Cells were counted in the AOB GC layer. The area of each examined section was measured with Neurolucida software (MicroBrightField, Colchester, VT, United States). Cell densities were calculated by summing cell counts made on all sections per animal and referred to the proper cellular layer volume (Σ of sampled areas × 25 μm). The percentage of double-labeled c-Fos/BrdU-positive cells, NeuN/BrdU-positive, GAD67/BrdU-positive and triple-labeled c-Fos/NeuN/BrdU-positive and c-Fos/GAD67/BrdU-positive in the AOB GC layer was established by counting labeled cells in parasagittal sections (25 μm thick) with a 40× objective with a confocal microscope using a UPI an FL 40× (N.A. 1.3) Olympus objective. One series (*n* = 3–4 sections/ animal) representing the whole AOB was used for each animal. In each section, all of the BrdU-positive cells were analyzed for co-expression with c-Fos, NeuN, GAD67, c-Fos/NeuN, or c-Fos/GAD67, and ratios of double-labeled or triple-labeled cells were determined. All cell counts were performed blind to the treatment. For statistical analysis, unpaired Student’s *t*-tests were used for simple 1:1 comparison of parametric data; paired Student’s *t*-test were used for sex and individual preference tests, in which same mice were measured twice, before (P20) and after (p41 or P50) puberty; one-way or two-way ANOVA were used for multiple comparisons for parametric data; chi-squared test was used for comparing the percentage of females showing lordosis.

### Sex Preference Tests

The preference of female mice for sex related odors was assessed by placing at opposite sides of the homecage (female subjects were either P20 or P41), urine stimuli were derived from adult male or female subjects (pooled from different individuals, 10 μl) on filter paper. The time spent investigating these stimuli was evaluated during 10 min trials.

### Learning of Familiar Male Odors

This assay evaluated the learned preference of a female mouse for male or female individual volatile scents that were previously experienced during a 5-day exposure period (familiarization), either with or without direct physical access to the odor source. This attractive response was based on an olfactory associative learning between volatile and non-volatile urinary components. During the exposure step, two urine stimuli obtained from distinct CD1 males (or females; 50 μl each) were placed on filter paper in the cage housing the female. While one of these filter papers was directly accessible to the female (leading to a conditioning learning of urine volatiles), the second one was placed in a meshed box to prevent direct physical contact with the odor source (thus preventing urine volatiles conditioning). Urine donors were adult CD1 males (or females; 4–5 months old) housed individually, with no kinship relation. Stimuli were delivered randomly two times a day each for 5 min within a total 4 h interval allowing for a 1 h resting phase between stimulations. After this learning period, a recognition step consisting of a two-choice olfactory preference test without physical contact was performed. Filter papers containing 50 μl of both directly (CS) or indirectly (US) previously experienced urine were deposited in meshed plastic boxes to prevent direct physical contact. During a 5 min trial period, stimulus investigation time was scored as the time spent in close contact with the stimulus source (distance of the snout from the box <1 cm) as well as the time spent manipulating, chewing, and biting the meshed box in an attempt to reach the stimulus source. As a critical control to rule out any pre-existing preference prior to the learning phase, we examined whether a given female showed a preference for volatiles in the tested urine sources; only females showing no preference were used for this assay.

### Female Sexual Receptivity (Lordosis)

Adult and juvenile female mice (*N* = 11 per group; both sexually naive) were single housed for 1 week and the estrus cycle was determined in order to use only animals in estrus or proestrus prior to the test. Adult males (sexually experienced) were introduced to the female’s home cage and were recorded for 1 h during the dark cycle. The number of lordosis events (in which females show a receptive still posture or arching of the back, allowing or promoting male mounting) by the females was assessed. The number of mounting behaviors as well as the latency to mount shown by male individuals were scored. The lordosis quotient was calculated as the ratio between lordosis events and male mounts, previously described as an index of female reproductive receptivity (Keller et al., 2006; Leinders-Zufall et al., 2014).

### SDS-PAGE

Mouse urine sample aliquots (2.5 μl) were mixed with 4x LDS buffer and 20x Reducing Reagent (Thermo Fisher Scientific, Waltham, MA, United States). Samples were then boiled and loaded on 4–12% SDS precast Bis-Tris NuPage gels (Thermo Fisher Scientific, Waltham, MA, United States). Gels were ran for 1 h at 170 V, fixed (50:10:40/methanol: acetic acid: H_2_O/ v:v:v), stained with Biosafe Coomassie (Bio-Rad, Hercules, CA, United States), and washed extensively with water. Gels were scanned using ChemiDoc MP System. Differences in protein content among samples was estimated by comparing gel band intensities using ImageJ.

## Results

### Male Odors Promote Granule Cell Survival in the AOB of Post-pubertal Females

Immature neurons migrating to the AOB typically reach a mature phenotype around 4 weeks after genesis (Oboti et al., 2009) and are particularly sensitive to olfactory stimulations after reaching the olfactory bulb (7 days after BrdU injection), between their 2nd and 3rd week (Oboti et al., 2011). They integrate mainly in the GC layer (Oboti et al., 2009, 2011), which is populated for the most part by GABAergic inhibitory neurons (GCs; Larriva-Sahd, 2008). Here, we further confirmed the fully mature phenotype of newborn GCs in the AOB 28 days after their genesis (91.58 ± 4.44% of BrdU-positive cells co-express NeuN, as in mature neurons; Mullen et al., 1992). In addition, we show that the vast majority of the BrdU-labeled neurons found in the AOB 4 weeks after genesis do have a GABAergic phenotype (84.51 ± 4.3% of BrdU-positive cells co-express GAD67, the GABA synthesis enzyme; Mugnaini et al., 1984; Supplementary Figure 1).

We previously found that exposure to male soiled bedding and male-derived urine compounds increases the survival of immature AOB GCs migrating from the SVZ in P50 female mice (a stage at which both brain circuits and neuroendocrine physiology can be considered mature; Finlay and Darlington, 1995), but not in pre-pubertal P20 females (Oboti et al., 2011). However, whether this effect is due to the different sensitivity of sensory circuits in the female brain or to other behavioral mechanisms (e.g., increase motivation in exploring these cues) affecting the female detection of male stimuli through the VNO, is not known.

**FIGURE 1:**
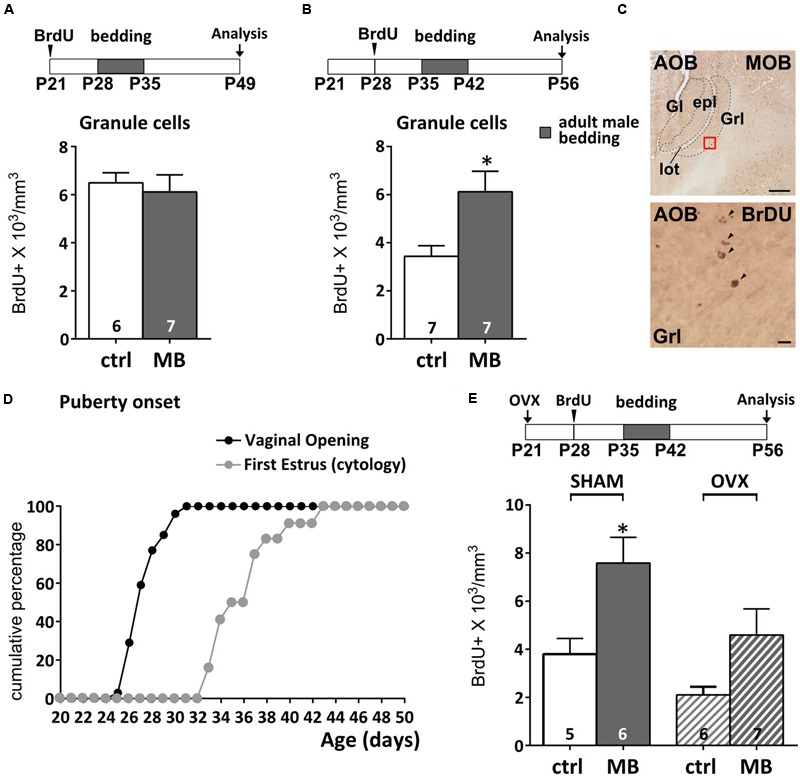
**Survival of newborn granule cells (GCs) in the accessory olfactory bulb (AOB) is sensitive to vomeronasal inputs after puberty. (A,B)** Daily exposure of female mice to male soiled bedding (MB) increases GC survival after the onset of the first estrus cycle (P35–P42). Bedding stimulations were timed during the 2nd week after GC genesis and their survival 28 dpi (unpaired Student’s *t*-test, P28–P35 ctrl vs. MB, *P* = 0.669, P35–P42 ctrl vs. MB, ^∗^*P* = 0.016). **(C)** Sagittal section of the AOB showing BrdU labeled nuclei (scale bar 100 μm). The lower panel refers to the red squared area in the GC layer (scale bar 5 μm). **(D)** Graph showing the onset of female puberty. This can be determined by examination of vaginal opening and the occurrence of the first estrus, assessed through cytological examination (see methods). **(E)** Bedding exposure in OVX pre-pubertal females (P21) does not modulate GC survival as significantly as in SHAM animals (two-way ANOVA, factors: ovariectomy, *F*_2,10_ = 6.766, *P* = 0.017; stimulus, *F*_2,10_ = 12.14, *P* = 0.002; interaction, *F*_2,10_ = 0.520, *P* = 0.479; Tukey’s *post hoc*, SHAM ctrl vs. SHAM MB, ^∗^*P* = 0.045; OVX ctrl vs. OVX MB, *P* = 0.206). Abbreviations: ctrl, control; MB, male bedding; MOB, main olfactory bulb; AOB, accessory olfactory bulb; Gl, glomerular layer; epl, external plexiform layer; GrL, granule cell layer; lot, lateral olfactory tract; OVX, ovariectomy; dpi, days post-injection. Sample sizes are indicated on each histogram bar. The values shown are the mean ± SEM.

To address this issue, we first sought to define more precisely the time at which AOB inhibitory circuits become sensitive to male bedding exposure. We analyzed the effect of 1 week male bedding stimulation on 2-weeks-old GCs (more sensitive to sensory activity; Oboti et al., 2011) birthdated by BrdU injections at different peripubertal stages: P21 and P28 (**Figures 1A,B**). BrdU systemic injection results in nuclear DNA labeling during cell division (Nowakowski et al., 1989) and therefore it has been used to localize newborn neurons at different times after genesis (**Figure 1C**). Following BrdU treatment, along with a Ca. 50% reduction in the basal level of AOB newborn neuronal survival (neurons born at P21 vs. P28), we found that exposure to adult male soiled bedding promotes the survival of newborn GCs in the AOB after P35 (**Figure 1B**), but not earlier (**Figure 1A**). This corresponds to the age of menarche, when the first estrus cycle occurs, as shown by vaginal patency assessment (opening of the external genitalia) and cytological examination (**Figure 1D**; Goldman et al., 2007). Around P27 almost 60% of the females showed vaginal opening, the 100% being reached only at about P30 (**Figure 1D**), similarly to what has been previously observed in both the CD1 (Szymanski and Keller, 2014) and C57 mouse strains (Nelson et al., 1990; Oboti et al., 2014). Signs of first estrus cycle occurrence were found only between 3 and 10 days later (50% of the females reached estrus around P35, while the rest reached estrus around P40–P42, similar to what occurring in C57 mice; **Figure 1D**; Nelson et al., 1990).

Puberty onset is accompanied by a surge of luteinizing hormone, follicle stimulating hormone and estrogens (Swerdloff and Odell, 1975) which affects both neuronal proliferation (e.g., prolactin, Menezes et al., 1995; Mak et al., 2007; Larsen et al., 2008; estrogens, Tanapat et al., 1999) and pubertal maturation (Koyama et al., 2013). Therefore, since exposure to male odor stimuli also modulates the production of ovarian hormones (Bronson and Maruniak, 1976), the survival of AOB GCs could depend on indirect hormone-mediated mechanisms, rather than VNO activity *per se*. Although male odor exposure was delivered when GCs were not further proliferating (7 days after genesis) but began to integrate in the AOB circuits (Luskin, 1993; Lois and Alvarez-Buylla, 1994), we could not rule out hormone-mediated effects on cell division occurring during late stages of migration through the rostral migratory stream (*rms*; Smith et al., 2001) or cell survival itself, which indeed have been previously reported (Veyrac and Bakker, 2011; Schellino et al., 2016).

We addressed this issue by performing the same sensory stimulations (from P35 to P42) in P28 BrdU-injected animals, after removal of the ovaries at P21. At this stage female gonadal hormones are not yet at peak (Bronson and Maruniak, 1976; Bronson, 1981). Therefore, 7 days after surgery, blood hormone levels remain stable at pre-pubertal levels (Bronson and Maruniak, 1976; Bronson, 1981). A two-way ANOVA indicated a significative effect of ovariectomy (OVX) on BrdU levels (regardless of bedding treatments). However, although OVX showed on average lower levels of BrdU expression in the AOB (**Figure 1E**), male bedding exposure in ovariectomized mice seemingly affected GC survival as in sham-operated subjects, however, without reaching statistical significance (see Supplementary Table 1 for statistical tests). Overall, these results suggest that gonadal hormones might modulate broad aspects of SVZ-OB neurogenesis (Menezes et al., 1995; Tanapat et al., 1999; Mak et al., 2007; Larsen et al., 2008; Brock et al., 2010; Brus et al., 2016), without critically affecting activity dependent modulation of GC survival (**Figure 1E**).

### Urine Odors from Mature, But Not Juvenile Males, Promote Granule Cell Integration

Male urine has been repeatedly shown to be efficient in promoting survival of AOB newborn cells in female mice (Oboti et al., 2009; Nunez-Parra et al., 2011; Schellino et al., 2016). In CD1 swiss mice, stimuli comprised in the low molecular weight (LMW) fraction of male urine (mass lower than 3 kDa) are more efficient in promoting neuronal integration of immature cells in the AOB of adult females (Oboti et al., 2011). This fraction contains volatile and non-volatile molecules resulting from the metabolism of hormones like testosterone (Knopf et al., 1983). Stimuli in the high molecular weight (HMW) fraction of male urine include instead proteins and large peptidic complexes, which production depends on both age and testosterone levels (Lombardi et al., 1976; Knopf et al., 1983; Chamero et al., 2007; Garratt et al., 2011). Therefore the composition of male chemostimuli varies with age, and urine protein content can be used as a proxy to assess these changes.

**FIGURE 2:**
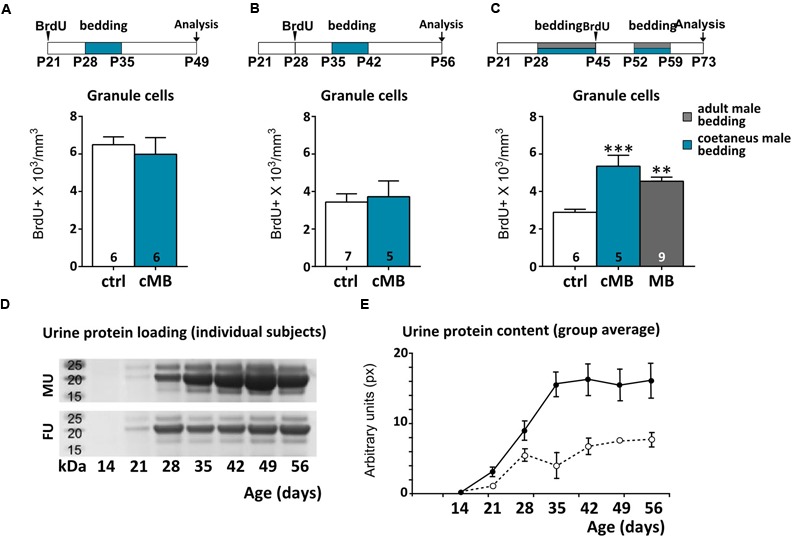
**Urine stimuli affecting AOB GCs in females are produced by sexually mature males. (A–C)** Comparison of the effect of coetaneous male bedding (cMB) stimulations delivered to female mice at different postnatal ages (P28, P35, P52), compared to mature male bedding (MB). Survival of AOB GCs is affected by odor exposures if the donors age is at least P52 (unpaired Student’s *t*-test, P28–P35 ctrl vs. cMB, *P* = 0.614, P35-P42 ctrl vs. cMB, *P* = 0.751; one-way ANOVA, *F*_2,19_ = 13.759, *P* = 0.000; Tukey’s *post hoc*, ctrl vs. cMB ^∗∗∗^*P* = 0.000; ctrl vs. MB, ^∗∗^*P* = 0.003). **(D)** PAGE of urine samples collected from male/female donors at different ages (P14–P56; the bands show the loading of proteins within the 15–27 kDa range, which includes MUPs). **(E)** Average protein content by age in the two sexes (males, solid line; females, dotted line; repeated measures two-way ANOVA, Supplementary Table 1). Abbreviations: MU, male urine; FU, female urine. Sample sizes are indicated on each histogram bar. The values shown are the mean ± SEM.

To clarify whether or not the maturity of male chemostimuli was differently affecting AOB GC density, we exposed female mice to bedding obtained from cages of coetaneous (cMB; i.e., same age of exposed females) but unrelated males. We found that such stimulation had no effect on BrdU-labeled GCs either at the time of vaginal opening (**Figure 2A**) or at the age of first estrus (1 week later; **Figure 2B**), when stimuli from mature males began to be effective (**Figure 1B**). Conversely, around P50, exposure to bedding material from coetaneous males (P50, cMB) increased the number of newborn GCs in the AOB as more mature stimuli do (**Figure 2C**). Thus, odor stimuli affected those cells generated right after these treatments (P45), even though females received daily bedding from the same donors (**Figure 2C**). Since long term exposure to the same odor stimuli has no reported effect on neuronal survival in the OB (Rochefort et al., 2002; Magavi et al., 2005), this suggests the occurrence of changes in the composition of male urine stimuli during peripubertal development. In addition, they also indicate that experience induced remodeling of AOB inhibitory circuits occurs in females at a time when donors (males) and receivers (females) of urine chemosignals are mature for mating. As a confirmation of reached sexual maturity in P50 males, we assessed the presence of urinary proteins, which production and excretion through urine are known to be testosterone dependent (Knopf et al., 1983; Chamero et al., 2007). We used gel electrophoresis to test individual urine samples collected from male CD1 mice at different ages. We found that at P50 male urine was already enriched with a high proportion of HMW compounds (15–25 kDa), while around P30 and younger ages urine protein content was lower, indicating a different composition of the stimuli (**Figures 2D,E**). Thus, male urine composition is likely to be a key factor affecting AOB GC survival, since female mice did not have previous experience with coetaneous donors in these experiments.

**FIGURE 3:**
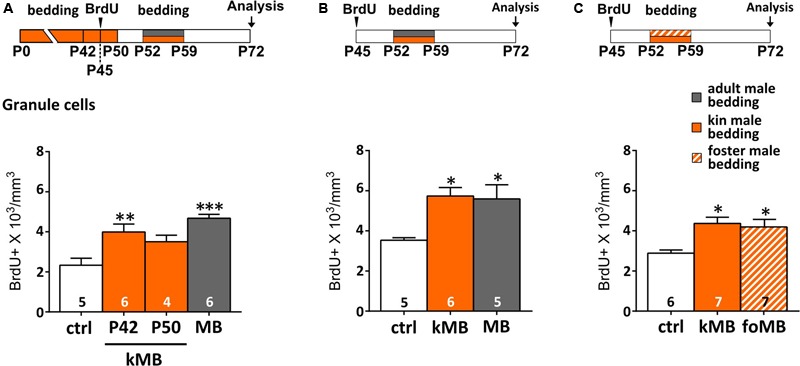
**Kinship does not prevent male odors to increase AOB GCs. (A)** Long-term exposure (until P42) to kin-related male stimuli (kMB) increases AOB GCs, after post-pubertal (P52–P59) exposure to the same stimuli, similarly to mature unrelated male cues (MB). By contrast, exposure to kMB cues until P50 does not increase AOB GCs density (one-way ANOVA, *F*_3,19_ = 10.47, *P* = 0.000; Tukey’s *post hoc*, ctrl vs. P42 kMB, ^∗∗^*P* = 0.008; ctrl vs. P50 kMB, *P* = 0.123, ctrl vs. MB, ^∗∗∗^*P* = 0.000). **(B)** Post-pubertal exposure to kin-related male stimuli (kMB) affects GC survival as do unrelated male odors (MB; one-way ANOVA, *F*_2,13_ = 6.508, *P* = 0.001; Tukey’s *post hoc*, ctrl vs. kMB ^∗^*P* = 0.02, ctrl vs. MB, ^∗^*P* = 0.03). **(C)** Post-pubertal stimulation (P52–P59) to bedding material soiled by foster littermates (foMB) is as effective as bedding soiled by kMB (one-way ANOVA, *F*_2,17_ = 6.493; *P* = 0.001; Tukey’s *post hoc*, ctrl vs. foMB ^∗^*P* = 0.024, ctrl vs. kMB, ^∗^*P* = 0.010). Sample size is indicated on each histogram bar. The values shown are the mean ± SEM.

Alternatively, early postnatal odor experience (nest odors, littermates) might have biased successive AOB male odor responses simply because the wiring of sensory neurons might have needed to retune (Xia et al., 2006; Jones et al., 2008; Hovis et al., 2012) to a new set of stimuli (e.g., MUPs and MHC-related compounds change with age, Singer et al., 1997; Sherborne et al., 2007). To verify this possibility, we have long-term exposed (P0–P42) female mice to the odors of kin-related coetaneous littermates, in order to evaluate GCs survival after puberty (as above). This was done by protracting bedding exposures a few days before (P42) or after (P50) BrdU injections (P45), in order to control for possible proliferative effects. We found that post-pubertal exposure to coetaneous littermates affected GC survival, similarly to what occurred when mature unrelated male stimuli are used (**Figure 3A**).

Specifically, the amount of BrdU-labeled GCs was significantly higher (compared to controls) when stimulations were interrupted 10 days before (P42) the second exposure (thus not covering the proliferative phase of cells labeled with BrdU at P45; **Figure 3A**). Conversely, lower levels of BrdU-labeling were found in the AOB GC layer when bedding stimulations were interrupted only 2 days before (P50) the second exposure (during the proliferative phase of BrdU-labeled cells; **Figure 3A**). This is in accordance to previous studies reporting a minor effect of long-term odor exposure on GCs survival (Magavi et al., 2005; Alonso et al., 2006). Kin-related odors affected GC survival in the AOB even when females were separated before puberty (P21; **Figure 3B**), thus confirming that the maturation of male stimuli is likely to be the most relevant factor affecting AOB plasticity. Finally, to rule out possible effects of donor genetic identity on GC survival in the AOB, we exposed mature females to male soiled bedding material from either kin-related (kMU) or unrelated foster cage mates (foMU), housed together with females until P21. Both stimuli were effective in modulating the survival of newborn GCs (**Figure 3C**). Overall, these results indicate that the survival of newborn GCs in the AOB of post-pubertal female mice is similarly affected by exposure to kin-related or unrelated males when they reach sexual maturity (P50).

### Differential Responses of AOB Circuits and Newborn Granule Cells to Familiar Odor Stimuli

**FIGURE 4:**
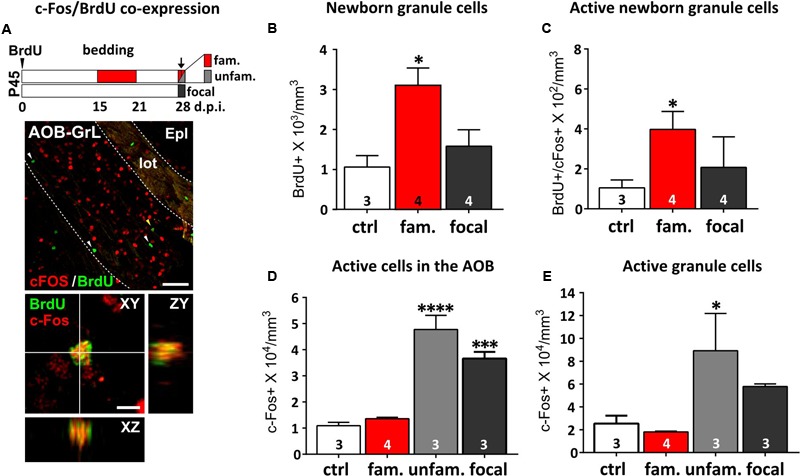
**Repeated exposure (familiarization) of females to male urine stimuli increases AOB newborn cell odor responses. (A)** On the top, protocol used for the analysis of AOB responsiveness to either familiar (fam; exposure to the same male bedding previously experienced from 15 to 21 dpi; red), unfamiliar (unfam; exposure to a different male bedding compared to the previously experienced; light gray) or focal odor exposures (without any previous experience; dark gray). Females were injected with BrdU at P45 and analyzed 28 dpi. On the bottom, co-expression of c-Fos (red) and BrdU (green) immunofluorescence in the AOB granule cell layer (GrL) measured 28 dpi (scale bar 50 μm). Arrowheads indicate c-Fos-negative/BrdU-positive cells. The yellow arrowhead indicates the c-Fos/BrdU double-stained cell magnified in the lower panel (scale bar 5 μm). **(B)** The BrdU-positive cell density measured in the AOB GC layer increases after male odor familiarization (fam), but not focal odor stimulations (focal; one-way ANOVA, *F*_2,8_ = 6.880, *P* = 0.018; Tukey’s *post hoc*, ctrl vs. fam, ^∗^*P* = 0.021). **(C)** Odor-evoked activity (c-Fos expression) in the BrdU-positive cells is higher after exposure to familiar stimuli (red), compared to focal stimulations and to novel odors (dark gray; unpaired Student *t*-test, ctrl vs. fam, ^∗^*P* = 0.046). **(D)** Evaluation of c-Fos expression in the entire cell population of the AOB after exposure of female mice to either familiar, unfamiliar or focal odor exposure (one-way ANOVA, *F*_3,9_ = 40.12, *P* = 0.0001; Tukey’s *post hoc*, ctrl vs. unfam ^∗∗∗∗^*P* = 0.000, ctrl vs. focal, ^∗∗∗^*P* = 0.001). **(E)** This effect is evident even considering the GC layer only (one-way ANOVA, *F*_3,9_ = 4.651, *P* = 0.0315; fam vs. unfam, Tukey’s *post hoc*, ^∗^*P* = 0.032). Sample sizes are indicated on each histogram bar. The values shown are the mean ± SEM.

Novel stimuli are shown to be most effective in promoting survival of newborn cells (Rochefort et al., 2002). Once odor stimulations are repeated through familiarization, newborn cells in the OB have been shown to preferentially respond to familiar odors, showing higher levels of c-Fos expression than pre-existing neurons (Magavi et al., 2005). Here, we reported similar effects occurring in the AOB (**Figures 4A,B**; Oboti et al., 2011). By exposing female mice to different male individual odors, either previously experienced (1 week odor familiarization) or novel, we evaluated odor-induced c-Fos expression in BrdU-positive GC neurons, when they are most responsive (i.e., around 2 weeks of age; **Figure 4A**; Oboti et al., 2011). Not only did 1 week of bedding exposure increase the amount of newborn GCs (BrdU-positive) in the AOB (**Figure 4B**), but also increased the percentage of those activated by familiar odors, as shown by a direct comparison between control group and familiarized females (c-Fos/BrdU co-expression; **Figure 4C**). Notably, c-Fos expression in AOB BrdU-positive cells was always associated to NeuN-positive/GAD67-positive inhibitory interneurons (Supplementary Figure 1). Interestingly, considering the entire AOB population, c-Fos activation in response to novel odors, in presence (unfamiliar) or absence (focal exposure) of previous familiarization, was always higher than the one evoked by either familiar odors or clean bedding (**Figure 4D**). This indicates that odor experience does not lead to a generalization in AOB responses to male cues but, instead, enhances the differences between different odor patterns, at least at the level of GCs (**Figure 4E**). Collectively, these results show that AOB immature GCs respond preferentially to familiar odor stimuli sensed during their integration into the local circuits, while the rest of the population shows increased responses to different novel stimuli, regardless of previous experience. Therefore, post-pubertal exposure to male odors increases the number of GCs responding to male odor cues and promote their integration in specific circuits implied in male odor responses.

### VNO-Dependent Identity Learning of Male Stimuli Is Displayed by Females after Puberty

Male urine odors sensed through the VNO exert higher activity levels in the female AOB, compared to female odors (Dudley and Moss, 1999; Kumar et al., 1999). In the AOB, neuronal survival was clearly affected by male odor stimuli 3 puberty, when these cues changed in composition (**Figures 2D,E**). However, the reasons why stimuli from adult males did not induce similar effects in the AOB of younger females were not clear. Since male cues are theoretically able to activate developing vomeronasal receptor neurons (Xia et al., 2006; Hovis et al., 2012; Oboti et al., 2015) and given that pre-pubertal hormone levels might not constrain eventual activity-dependent effects on AOB GC survival (**Figure 1E**), we hypothesized the presence of behavioral mechanisms limiting the contact of peripubertal female mice to adult male cues.

**FIGURE 5:**
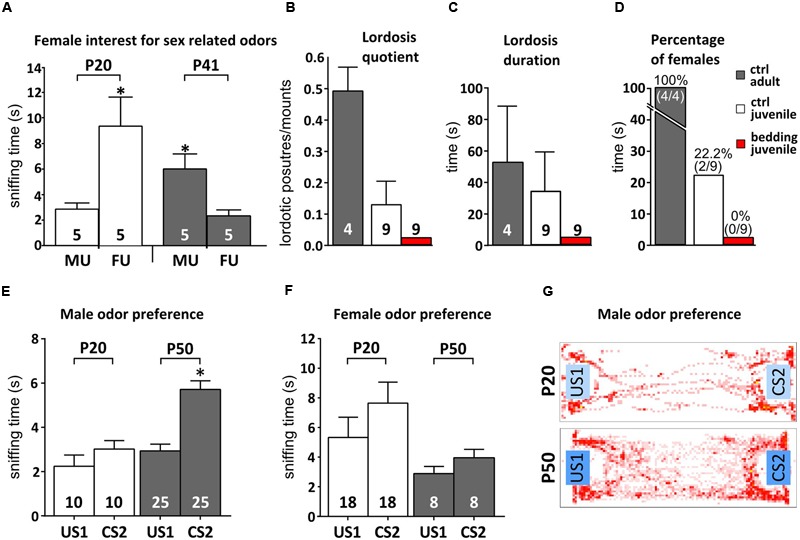
**Female mice learn to prefer familiar male odors after puberty. (A)** Reversal of female preference for sex odors (MU, male urine; FU, female urine) across puberty (P20 and P41; unpaired Student’s *t*-test, P20 MU < FU, ^∗^*P* < 0.05; P41 MU > FU, ^∗^*P* < 0.05). **(B–D)** Evaluation of female receptivity (lordosis quotient, duration and percentage of females displaying lordosis) after peripubertal exposure to male soiled bedding compared to unstimulated controls (ctrl; **B**- one-way ANOVA, *F*_2,18_ = 6.386, *P* = 0.008; Tukey’s *post hoc*, juv ctrl vs. juv bed *P* = 0.402, juv ctrl vs. adult ctrl, *P* = 0.048, juv bed vs. adult ctrl, *P* = 0.006; **C**- one-way ANOVA, *F*_2,18_ = 1.672, *P* = 0.217; Tukey’s *post hoc*, juv ctrl vs. juv bed *P* = 0.383, juv ctrl vs. adult ctrl, *P* = 0.842, juv bed vs. adult ctrl, *P* = 0.244; **D**- chi-squared contingency test, χ^2^(2, *N* = 22) = 14.16, *P* = 0.008). Adult levels were calculated in P90 mature female mice. **(E)** Male odor preference in post-pubertal females after exposure to volatiles male cues. Volatile odors become preferred after repeated direct contact to non-volatile male stimuli (conditioned stimuli, CS) in respect to never experienced volatile odors (unconditioned stimuli, US). The graph shows an increase in preference for volatile male odors (CS2) after familiarization. Female mice develop this preference only after puberty (as P20 females show equal responses to CS and US stimuli; unpaired Student *t*-test, P50 CS2 > US1, ^∗^*P* < 0.05). **(F)** Exposure to female odors in the same behavioral paradigm does not lead to preference learning. **(G)** Heatmaps representing the mouse presence in the cage during behavioral tests. Sample sizes are indicated on each histogram bar. The values shown are the mean ± SEM.

We tested this idea by first measuring the time spent by mixed housed female mice to investigate odors from both adult males and females, before (P20) and after (P41) puberty onset. While younger females spent more time investigating female odors (paired Student’s *t*-test, *P* = 0.029), after the onset of puberty this preference is reversed, with P40 females displaying higher levels of investigation toward male cues (paired Student’s *t*-test, *P* = 0.025; **Figure 5A**). This implies that juvenile females might assign a different meaning to male odors, if compared to reproductively mature subjects. Therefore, it could be that male odor stimuli have an impact on neuronal survival in the AOB not only when they acquire a mature composition but also when they become meaningful mating signals. Since male odors promote female receptivity during mating (Jemiolo et al., 1991; Ramm et al., 2008; Haga et al., 2010; Roberts et al., 2010), we addressed this point by evaluating the impact of peripubertal male odor exposure on female lordosis behavior toward male intruders. Female mice were exposed to male soiled bedding from P35 to P42, prior to behavioral tests. In general, juvenile females (P43–P45) showed low receptivity toward adult male intruders (**Figures 5B–D** and relative statistics in Supplementary Table 1) as compared to adults, either in absence of any male stimuli, or after 1 week daily exposure to male urine, further supporting that at this age male odors do not represent mating signals promoting female receptivity. Nonetheless, given the role of AOB GCs in shaping mitral cell odor selectivity (Hendrickson et al., 2008), we hypothesized that the addition of new GCs to pre-existing AOB circuits might occur when the vomeronasal sensory neurons begins to tune their discriminative capabilities at puberty. In order to test this possibility, we quantified the female learned preference for individual male volatile odors: after repeated direct exposure to individual male chemostimuli, female mice prefer to investigate the volatile cues derived from the same male, if compared to other subjects. This selective preference is established through short-term experience of urine derived cues sensed through the vomeronasal pathway (Moncho-Bogani et al., 2002; Ramm et al., 2008; Hurst, 2009; Oboti et al., 2014). Accordingly, we found that repeated direct exposure to male individual urine compounds induced in post-pubertal females resulted in a higher interest toward the associated volatile stimuli, as compared to unexperienced odors (**Figures 5E,G**). Conversely, pre-pubertal exposure to male odors failed to elicit this effect in female mice (**Figures 5E,G**). However, exposure to mature female odors, had negligible effects in both juvenile and mature females (**Figure 5F**), indicating sex-specificity in this response. Overall, these results suggest that male stimuli used to establish male individual preference (or simply discrimination of experienced individual odors) may not gain efficient access to the VNO sensory epithelium until more mature stages.

**FIGURE 6:**
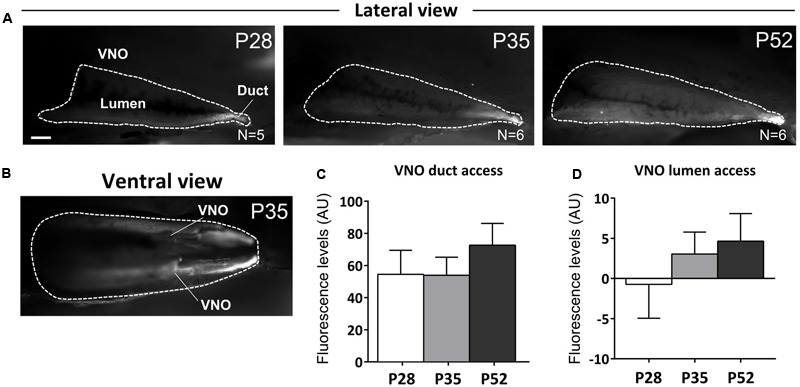
**Vomeronasal organ intake of male urine derived stimuli increases after puberty. (A,B)** Sagittal dissection of the nasal cavity showing rhodamine intake in the VNO upon nostril presentation of dyed-male urine to female mice at different postnatal ages (P28, P35, P52; **A**-lateral view, **B**-ventral view; scale bar 0.5 mm). Fluorescent labeling inside the VNO indicates active stimulus intake in the VNO during investigation. **(C,D)** Fluorescence levels (AU), measured at the level of the VNO duct **(C)** and internal lumen **(D)** indicate appropriate stimulus delivery at all three postnatal ages (P28, P35, and P52) and increased levels of stimulus intake (see text for major details). Abbreviations: AU, arbitrary units. The values shown are the mean ± SEM.

To clarify this point, we measured the access of male urine stimuli to the VNO of female mice at different peripubertal ages (P28, P35, and P52). To evaluate the extent up to which male cues reach the VNO epithelia in females, male urine was mixed with a fluorescent dye (rhodamine, 0.002%, Ogura et al., 2010). We found a positive correlation between rhodamine-based fluorescence and age (P28–P52), in both the VNO duct (*R*^2^ = 0.726) and lumen (*R*^2^ = 0.948), suggesting either an increased access or intake of male derived cues to the VNO sensory epithelia [no significant differences were evident using one-way ANOVA, see Supplementary Table 1; duct *F*_(2,14)_ = 0.674, *P* = 0.526; lumen *F*_(2,14)_ = 0.609, *P* = 0.558]. At younger stages, rhodamine fluorescence was mainly present in the duct at the entrance of the VNO while at later stages, fluorescence was clearly visible across the whole extent of the inner lumen of the VNO (**Figures 6A–D**). We evaluated male urine intake bilaterally and analyzed rhodamine fluorescence in both VNOs (**Figure 6B**). In both the VNO duct and the lumen of the sensory epithelium, there was an increased tendency of rhodamine retention across puberty (by measuring fluorescence in arbitrary units ranged between 0 and 256, the maximum values were observed at older ages P35, P52), according to a delayed patency of the duct giving access to the VNO (Coppola et al., 1993). Overall these results suggest that the detection of male urine compounds through the VNO might increase during female puberty and therefore promote the addition of newborn GCs to AOB circuits involved in individual odor processing.

## Discussion

Previously, we have shown that survival of AOB inhibitory interneurons is modulated by sensory activity only in post-pubertal female mice (Oboti et al., 2009, 2011). Here, we aimed to explore the physiological and behavioral parameters underlying the delayed sensitivity of GC survival to male urine odors. First, we show that male bedding exposure affects GCs at the onset of the first estrus cycle. Male stimuli produced by sexually mature males are more effective than stimuli from younger males. Moreover, the genetic identity of the donors does not affect GC survival, as kin-related and unrelated stimuli induced similar effects. The pubertal onset of GC sensitivity to male odor cues is unlikely mediated by hormonal changes occurring in females at this stage, although gonadal hormones might affect neuronal survival indirectly, by regulating VNO pumping activity and sensory detection (Dey et al., 2015), or by modulating either cell proliferation or migration in the SVZ as previously reported (Menezes et al., 1995; Tanapat et al., 1999; Mak et al., 2007; Larsen et al., 2008). Conversely, this timing correlated with a higher intake of male signals by the female VNO, observed from the onset of puberty onward, therefore directly linking the maturation of AOB inhibitory circuits to olfactory sensory activity. Consistently, after short-term exposure to male odors, newborn GCs showed higher odor specific responses. As a consequence of this experience, male cues become preferred over unfamiliar or novel stimuli (Ramm et al., 2008). Overall, our results indicate that the first brain relay of the VNS, the AOB, is capable of rearranging its circuits by increasing the survival of newborn GCs in response to environmental stimuli once they become meaningful social signals during peripubertal development.

Puberty represents a phase in which the meaning of social signals is subjected to changes, due to the increased complexity of the behavioral contexts in which such signals are used (mating, territoriality, parental care). Because both the identity and the relative composition of social signals might be subject to contingent variation (Sappington and Taylor, 1990; Clark et al., 1997; Moore, 1997; Mucignat-Caretta et al., 1998; Garratt et al., 2011), a reliable way to communicate information about social and reproductive status should imply a certain degree of adaptation in order to optimize the correspondence between chemical signals and behavioral response (Lemmen and Evenden, 2009; Villar et al., 2015). Neuronal turnover in other OB inhibitory networks has long been proposed to meet this demand by optimizing mitral cell odor representations (Alonso et al., 2006; Lepousez et al., 2014). Consistently, odor-evoked activity affects both the maturation and the integration of immature interneurons depending on the extent and value of odor experience (Rochefort et al., 2002; Magavi et al., 2005; Lledo et al., 2006; Schellino et al., 2016). Nonetheless, in most of the functional studies focused on the VNS, this plasticity has been often disregarded (but see for example Imayoshi et al., 2008; Sakamoto et al., 2011, 2014). The main reason for this is the misconception that animal social behaviors must be somehow constrained by the presence of highly conserved molecular signals and highly specialized olfactory receptors to which they bind. In this scenario, increasing behavioral complexity necessarily requires a concurrent increase in the number of signals used to match behavioral needs to behavioral responses (for example adult sexual interactions are promoted by the peptide ESP1 and adult-juvenile interactions inhibited by ESP22; Haga et al., 2010; Ferrero et al., 2013). However, the presence of redundancy in both signal production and detection (Marino et al., 2000) provides evidence that single molecules alone cannot be reliable signals in social communication.

For instance, different molecules are used as puberty accelerating signals (DHT, DHB, MUPs: Novotny et al., 1999a; Mucignat-Caretta et al., 1995), signals for triggering aggression (MUPs, DHT, DHB: Novotny et al., 1985; Chamero et al., 2007) or signals for promoting female attraction (Darcin, ESP1, MUPs: Kimoto et al., 2007; Haga et al., 2010; Roberts et al., 2010; Oboti et al., 2014). Therefore, in the case of MUPs for example, the signaling properties depend probably more on the receiver’s behavioral state, rather than on the specific ligand/receptor interactions.

Given the high affinity of VNO receptors to their ligands, it would be both evolutionarily and metabolically expensive if this olfactory system were to maintain the potential to detect all potential ligands for its receptors. Our results show that the activation of AOB granules depends on the identity of experienced stimuli. Importantly, the inhibitory feedback they provide to AOB mitral cells is responsible for the highly selective responses shown by these output neurons to individual odors (Luo et al., 2003; Hendrickson et al., 2008; Ben-Shaul et al., 2010). Therefore, the activity dependent plasticity of AOB GCs potentially represents a mechanism by which such odor specific responses are established through experience. Eventually, this further implies that social behavioral displays triggered by the VNS might involve a certain degree of adaptation to signal availability occurring through experience. However, future experiments are necessary to define both the contexts and the constraints of such plasticity.

Importantly, we do not want to argue that the VNO is not functional before puberty, as several reports indicated that pre-pubertal animals are indeed sensitive to stimuli detected through vomeronasal sensory neurons (Lomas and Keverne, 1982; Nishimura et al., 1989; Price and Vandenbergh, 1992; Mucignat-Caretta et al., 1995; Novotny et al., 1999a,b; Mucignat et al., 2004; Hovis et al., 2012). Despite juvenile (pre-pubertal) female mice are probably able to sense male odorants through the VNO, they are likely to be less motivated to investigate them since male odors are repulsive for pre-pubertal females (Mucignat-Caretta et al., 1998). However, although these stimuli might affect the advancement of female puberty onset (Lomas and Keverne, 1982; Price and Vandenbergh, 1992; Novotny et al., 1999a,b; Jouhanneau et al., 2014), at this stage they might still be ineffective in promoting sexual receptivity, as shown by our behavioral assays. This is probably because, both behaviorally and physiologically, female mice might be still immature at this stage. In addition, it has been shown that the patency of the vomeronasal duct matures progressively during postnatal development and around puberty (Coppola et al., 1993), suggesting the occurrence of changes in the general physiology of the VNS as well as in the female reproductive physiology, in a phase in which male chemical cues acquire the meaning of sexual signals utilized in the context of mating and reproductive behaviors. All these factors might be equally relevant in explaining the post-pubertal sensitivity of AOB GC survival to sensory activity.

Overall, our results provide evidence in support of the pubertal activity-dependent tuning in sensory circuits conveying salient signals for mating and social behaviors, as proposed for higher order brain circuits as well (see for a review Piekarski et al., 2017). Moreover, they corroborate the idea that the functional maturation of the neural systems implied in goal-oriented behaviors might occur not only through modulatory mechanisms activated by hormones and neurotransmitters (see for example Anderson, 2016), but also through activity-dependent changes in the wiring of the underlying circuits (as shown also by Jones et al., 2008; Hovis et al., 2012).

## Author Contributions

LO, ST, and PP designed and planned the experiments. ST, LO, and RS performed the experiments. LO and ST analyzed the data. NH, OA, and WL designed and performed the VNO-dye assay and analyzed the data. ST and MM performed ovariectomies. MS performed the gel electrophoresis experiment. ST prepared the figures of the paper. LO wrote the manuscript.

## Conflict of Interest Statement

The authors declare that the research was conducted in the absence of any commercial or financial relationships that could be construed as a potential conflict of interest.
