# Supplementary material for: Activity Dependent Modulation of Granule Cell Survival in the Accessory Olfactory Bulb at Puberty
Source: Front Neuroanat. 2017 May 23;11:44. doi: 10.3389/fnana.2017.00044 (PMC5440572; doi:10.3389/fnana.2017.00044)
Supplement: Supplementary file 1 [file Table_1.PDF]

Supplementary Material

Activity Dependent Modulation of Granule Cell Survival in the Accessory Olfactory Bulb at Puberty

Oboti L.*, Trova S., Peretto P. *

*** Correspondence:** Corresponding Author: Livio Oboti, livio.oboti@gmail.com; Paolo Peretto, paolo.peretto@unito.it

1. **Supplementary Table 1.**

FIGURE	*Experimental Paradigm*	GROUPS	STATISTICAL TEST	F value	P value
**1A** AOB Grl BrdU+ cells	*P28-P35 bedding exp*	ctrl *vs* MB	Unpaired Student's *t*-test		*P*=0.669
**1B** AOB Grl BrdU+ cells	*P35-P42 bedding exp*	ctrl *vs* MB	Unpaired Student's *t*-test		*P*=0.016
**1E** AOB Grl BrdU+ cells	*P35-P42 bedding exp*	SHAM ctrl *vs* SHAM MB *vs* OVX ctrl *vs* OVX MB	Two-way ANOVA		
			Ovariectomy	$F_{(1,20)}$=6.766	*P*=0.017
			Stimulus	$F_{(1,20)}$=12.14	*P*=0.002
			Interaction	$F_{(1,20)}$=0.520	*P*=0.479
		SHAM ctrl *vs* SHAM MB	Tukey post hoc		*P*=0.045
		SHAM ctrl *vs* OVX ctrl	Tukey post hoc		*P*=0.587
		SHAM ctrl *vs* OVX MB	Tukey post hoc		*P*=0.924
		SHAM MB *vs* OVX ctrl	Tukey post hoc		*P*=0.002
		SHAM MB *vs* OVX MB	Tukey post hoc		*P*=0.098
		OVX ctrl *vs* OVX MB	Tukey post hoc		*P*=0.206
**2A** AOB Grl BrdU+ cells	*P28-P35 bedding exp*	ctrl *vs* cMB	Unpaired Student's *t*-test		*P*=0.614
**2B** AOB Grl BrdU+ cells	*P35-P42 bedding exp*	ctrl *vs* cMB	Unpaired Student's *t*-test		*P*=0.751
**2C** AOB Grl BrdU+ cells	*P28-P45, P52-P59 bedding exp*	ctrl *vs* MB *vs* cMB	One-way ANOVA	$F_{(2,17)}$=13.76	*P*=0.0003
		cMB *vs* MB	Tukey post hoc		*P*=0.204
		ctrl *vs* MB	Tukey post hoc		*P*=0.003
		ctrl *vs* cMB	Tukey post hoc		*P*=0.000
**2E** urine protein content		P14 *vs* P21 *vs* P28 *vs* P35 *vs* P42 *vs* P49 *vs* P56<br>Males *vs* Females	Repeated measures Two-way ANOVA		
			Postnatal days	$F_{(6,24)}$=15.67	*P*<0.0001
			Gender	$F_{(1,4)}$=5.737	*P*=0.075
			Interaction	$F_{(6,24)}$=3.306	*P*=0.016
	Males	P14 *vs* P21	Bonferroni post hoc		*P*>0.999
	SDS-PAGE	P14 *vs* P28			*P*=0.366

**2E** urine protein content	*Males* *SDS-PAGE*	P14 *vs* P35		*P*=0.001
		P14 *vs* P42		*P*<0.0001
		P14 *vs* P49		*P*<0.0001
		P14 *vs* P56		*P*=0.004
		P21*vs* P28		*P*>0.999
		P21*vs* P35		*P*=0.003
		P21*vs* P42		*P*<0.0001
		P21*vs* P49		*P*<0.0001
		P21*vs* P56		*P*=0.015
		P28 *vs* P35		*P*=0.379
		P28 *vs* P42		*P*=0.004
		P28 *vs* P49		*P*=0.006
		P28 *vs* P56		*P*>0.999
		P35 *vs* P42		*P*>0.999
		P35 *vs* P49		*P*>0.999
		P35 *vs* P56		*P*>0.999
		P42 *vs* P49		*P*>0.999
		P42 *vs* P56		*P*=0.404
		P49 *vs* P56		*P*=0.526
**2E** urine protein content	*Females* *SDS-PAGE*	P14 *vs* P21	Bonferroni post hoc	*P*>0.999
		P14 *vs* P28		*P*=0.976
		P14 *vs* P35		*P*>0.999
		P14 *vs* P42		*P*=0.113
		P14 *vs* P49		*P*=0.026
		P14 *vs* P56		*P*=0.236
		P21 *vs* P28		*P*>0.999
		P21 *vs* P35		*P*>0.999
		P21 *vs* P42		*P*>0.999
		P21 *vs* P49		*P*=0.407
		P21 *vs* P56		*P*>0.999
		P28 *vs* P35		*P*>0.999
		P28 *vs* P42		*P*>0.999
		P28 *vs* P49		*P*>0.999
		P28 *vs* P56		*P*>0.999
		P35 *vs* P42		*P*>0.999
		P35 *vs* P49		*P*>0.999
		P35 *vs* P56		*P*>0.999
		P42 *vs* P49		*P*>0.999
		P42 *vs* P56		*P*>0.999
		P49 *vs* P56		*P*>0.999

**3A** AOB Grl BrdU+ cells	*P0-P42/P50, P52-P59* *bedding exp*	ctrl *vs* p42 kMB *vs* p50 kMB *vs* MB	One-way ANOVA	$F_{(3,19)}$=10.47	*P*=0.0003
		ctrl *vs* p42 kMB	Tukey post hoc		*P*=0.008
		ctrl *vs* p50 kMB	Tukey post hoc		*P*=0.123
		ctrl *vs* MB	Tukey post hoc		*P*=0.000
		p42 kMB *vs* p50 kMB	Tukey post hoc		*P*=0.750
		p42 kMB *vs* MB	Tukey post hoc		*P*=0.345
		p50 kMB *vs* MB	Tukey post hoc		*P*=0.081
**3B** AOB Grl BrdU+ cells	*P52-P59* *bedding exp*	ctrl *vs* kMB *vs* MB	One-way ANOVA	$F_{(2,13)}$=6.508	*P*=0.001
		ctrl *vs* kMB	Tukey post hoc		*P*=0.015
		ctrl *vs* MB	Tukey post hoc		*P*=0.028
		kMB *vs* MB	Tukey post hoc		*P*=0.976
**3C** AOB Grl BrdU+ cells	*P52-P59* *bedding exp*	ctrl *vs* kMB *vs* foMB	One-way ANOVA	$F_{(2,17)}$=6.493	*P*=0.001
		ctrl *vs* kMB	Tukey post hoc		*P*=0.010
		ctrl *vs* foMB	Tukey post hoc		*P*=0.024
		kMB *vs* foMB	Tukey post hoc		*P*=0.903
**4B** AOB Grl BrdU+ cells	*P60-P66* *bedding exp*	ctrl *vs* fam *vs* focal	One-way ANOVA	$F_{(2,8)}$=6.880	*P*=0.018
		ctrl *vs* fam	Tukey post hoc		*P*=0.021
		ctrl *vs* focal	Tukey post hoc		*P*=0.664
		fam *vs* focal	Tukey post hoc		*P*=0.056
**4C** AOB Grl BrdU/cFOS cells	*P60-P66* *bedding exp*	ctrl *vs* fam *vs* focal	One-way ANOVA	$F_{(2,8)}$=1.623	*P*=0.256
		ctrl *vs* fam	Tukey post hoc		*P*=0.250
		ctrl *vs* focal	Tukey post hoc		*P*=0.820
		fam *vs* focal	Tukey post hoc		*P*=0.474
		ctrl *vs* fam	Student *t*-test		*P*=0.046
**4D** AOB cFOS cells	*P60-P66* *bedding exp*	ctrl *vs* fam *vs* unfam*VS* focal	One-way ANOVA	$F_{(3,9)}$=40.12	*P*<0.0001
		ctrl *vs* fam	Tukey post hoc		*P*=0.901
		ctrl *vs* unfam	Tukey post hoc		*P*<0.0001
		ctrl *vs* focal	Tukey post hoc		*P*=0.001
		fam *vs* unfam	Tukey post hoc		*P*<0.0001
		fam *vs* focal	Tukey post hoc		*P*=0.001
		unfam *vs* focal	Tukey post hoc		*P*=0.093
**4E** AOB Grl cFOS cells	*P60-P66* *bedding exp*	ctrl *vs* fam *vs* unfam *vs* focal	One-way ANOVA	$F_{(3,9)}$=4.651	*P* =0.032
		ctrl *vs* fam	Tukey post hoc		*P*=0.984
		ctrl *vs* unfam	Tukey post hoc		*P*=0.074
		ctrl *vs* focal	Tukey post hoc		*P*=0.500
		fam *vs* unfam	Tukey post hoc		*P*=0.032

		fam *vs* focal	Tukey post hoc		*P*=0.291
		unfam *vs* focal	Tukey post hoc		*P*=0.525
**5A** sniffing time (s)	*Female sex odors preference*	P20 FU > MU	Paired Student's*t*-test		*P*=0.03
		P41 FU < MU	Paired Student's*t*-test		*P*=0.03
**5B** lordotic postures/ mounts	*Lordosis quotient*	juv ctrl *vs* juv bed *vs* adult ctrl	One-way ANOVA	$F_{(2,18)}$=6.386	*P*=0.008
		juv ctrl *vs* juv bed	Tukey post hoc		*P*=0.402
		juv ctrl *vs* adult ctrl	Tukey post hoc		*P*=0.048
		juv bed *vs* adult ctrl	Tukey post hoc		*P*=0.006
**5C** time(s)	*Lordosis duration*	juv ctrl *vs* juv bed *vs* adult ctrl	One-way ANOVA	$F_{(2,17)}$=1,672	*P*=0.217
		juv ctrl *vs* juv bed	Tukey post hoc		*P*=0.383
		juv ctrl *vs* adult ctrl	Tukey post hoc		*P*=0.842
		juv bed *vs* adult ctrl	Tukey post hoc		*P*=0.244
**5D** Lordosis expression	*Percentage of females showing lordosis*	juv ctrl *vs* juv bed *vs* adult ctrl	Chi-square	χ^2(2, N=22) = 14,16	*P*=0.0008
**5E** sniffing time (s)	*Male odor preference*	P20 US1 = CS2	Unpaired Student's*t*-test		*P*>0.05
		P20 US1 < CS2	Unpaired Student's*t*-test		*P*<0.05
**5F** sniffing time (s)	*Female odor preference*	P20 US1 = CS2	Unpaired Student's*t*-test		*P*>0.05
		P20 US1 = CS2	Unpaired Student's*t*-test		*P*>0.05
**6C** fluorescence levels (AU)	*VNO Duct*	P28 *VS* P35 *VS* P52	One-way ANOVA	$F_{(2,14)}$=0.674	*P*=0.526
		P28 *VS* P35	Tukey post hoc		*P*=0.999
		P28 *VS* P52	Tukey post hoc		*P*=0.615
		P35 *VS* P52	Tukey post hoc		*P*=0.568
**6D** fluorescence levels (AU)	*VNO Lumen*	P28 *VS* P35 *VS* P52	One-way ANOVA	$F_{(2,14)}$=0.609	*P*=0.558
		P28 *VS* P35	Tukey post hoc		*P*=0.731
		P28 *VS* P52	Tukey post hoc		*P*=0.539
		P35 *VS* P52	Tukey post hoc		*P*=0.939
**6C** fluorescence levels (AU)	*VNO Duct*		Regression analysis		R^2=0.726
**6D** fluorescence levels (AU)	*VNO Lumen*		Regression analysis		R^2=0.948
