# Supplementary material for: Activity Dependent Modulation of Granule Cell Survival in the Accessory Olfactory Bulb at Puberty
Source: Front Neuroanat. 2017 May 23;11:44. doi: 10.3389/fnana.2017.00044 (PMC5440572; doi:10.3389/fnana.2017.00044)
Supplement: Supplementary file 2 [file Image_1.PDF]

Supplementary Material

Activity Dependent Modulation of Granule Cell Survival in the Accessory Olfactory Bulb at Puberty

Oboti L.*, Trova S., Peretto P. *

*** Correspondence:** Corresponding Author: Livio Oboti, livio.oboti@gmail.com; Paolo Peretto, paolo.peretto@unito.it

1. **Supplementary Figures**

Supplementary Figure 1.

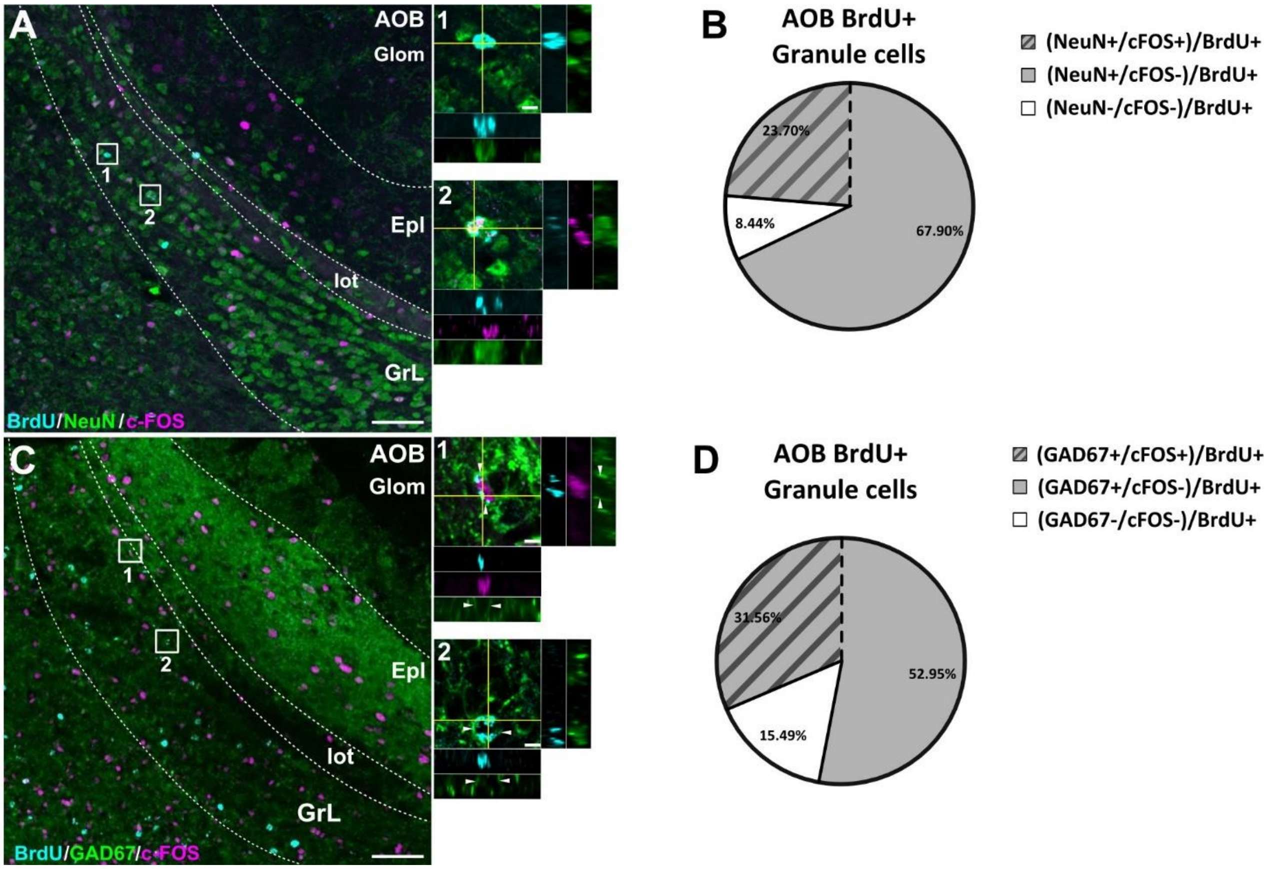

Supplementary Figure 1. A) Immunostaining of BrdU (cyan), NeuN (green) and cFos (magenta) immunofluorescence in the AOB granule cell layer (GrL) evaluated 28 d.p.i. (scale bar 50µm). Magnification panel 1 represents a c-Fos-negative/NeuN-positive/BrdU-positive granule cell and panel 2 a c-Fos-positive/NeuN-positive/BrdU-positive cell (scale bars 5µm). **B)** Percentage of newborn (BrdU) granule cells expressing NeuN (grey). Stripes indicate the percentage of newborn NeuN-positive cells activated (c-Fos-positive) by bedding exposure. **C)** Co-expression of BrdU (cyan), GAD67 (green) and cFos (magenta) immunofluorescence in the AOB granule cell layer (GrL) measured 28 d.p.i. (scale bar 50µm). Magnification panel 1 represents a c-Fos-positive/GAD67-positive/BrdU-positive granule cell and panel 2 a c-Fos-negative/NeuN-positive/BrdU-positive cell (scale bars 5µm). **D)** Percentage of newborn (BrdU) granule cells expressing GAD67 (grey). Stripes indicate the percentage of newborn GAD67-positive cells activated (c-Fos-positive) by bedding exposure. N=3 animals per group.
